# Thermal spin transport phenomena and their correlation to magnetic properties of metallic Pt/Co_1-x_Fe_x_ and Pt/Ni_1-x_Fe_x_ bilayers

**DOI:** 10.1080/14686996.2025.2587389

**Published:** 2025-11-10

**Authors:** Panagiota Bougiatioti, Orestis Manos, Günter Reiss, Timo Kuschel

**Affiliations:** a Faculty of Physics, Bielefeld University, Bielefeld, Germany; b Institute of Physics, Johannes Gutenberg University Mainz, Mainz, Germany

**Keywords:** Spin Seebeck effect, anomalous Nernst effect, magnetic proximity effect

## Abstract

We investigate the thermal spin transport phenomena in ferromagnetic metals with an adjacent spin-polarized Pt layer examining sputter-deposited Pt/Co_1-x_Fe_x_ and Pt/Ni_1-x_Fe_x_ bilayers. We quantitatively disentangle the detected voltages generated by the spin Seebeck effect from the anomalous Nernst effect contributions arising from both the ferromagnetic metal and the spin-polarized Pt layer. Further, we probe the dependence of the aforementioned effects on the composition and on the magnetic moments of both the ferromagnetic metal and the spin-polarized Pt layer. We report a strong dependence of all effects on the composition via increase/decrease of the effect coefficients with increasing/decreasing magnetic moments of both the ferromagnetic metal and the spin-polarized Pt layer. Following our descriptions, our work provides quantitative spin Seebeck coefficients in metals and thermal spin transport coefficients in nominally non-magnetic materials such as Pt which will be the base for future designs of spin caloritronic applications.

## IMPACT STATEMENT

We explored the longitudinal spin Seebeck effect (LSSE) in metallic systems and disentangled it from anomalous Nernst contributions. So far, most of the reports on LSSE have investigated magnetic insulators. The systematic study of thermally induced spin transport in Pt/Ni_1-x_Fe_x_ and Pt/Co_1-x_Fe_x_ with the variation of the Fe content x has not been studied so far. In addition, we identified the magneto-thermal effect in the spin-polarized Pt layers that are magnetized due to the magnetic proximity effect. The systematic variation of the proximity-induced anomalous Nernst effect by the magnetic moment in the ferromagnetic support and, thus, the magnetic proximity strength has not been subject of research studies so far. Our quantitative analysis and separation of spin Seebeck effect, proximity-induced anomalous Nernst effect and anomalous Nernst effect in the feromagnet will help to design new material combinations for efficient spin caloritronic devices.

## GRAPHICAL ABSTRACT

**Figure:**
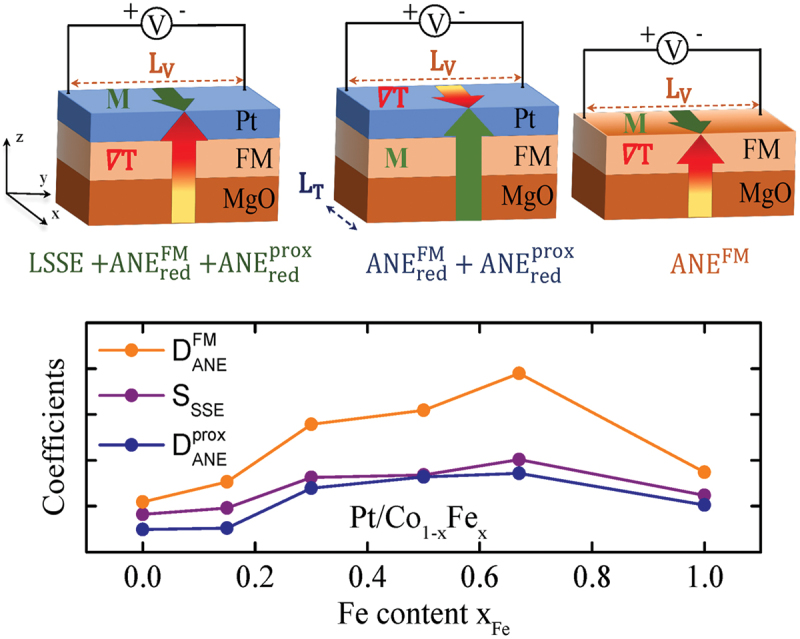

## 1. Introduction

The established field of spin caloritronics combines research on spin-related phenomena with thermoelectric effects [1–3]. This research field has been initiated by the discovery of the spin Seebeck effect (SSE) [4]. Its major importance relies on the efficient generation of a spin current induced by an applied temperature gradient as a spin analogy of the conventional Seebeck effect [5]. In particular, when a temperature gradient is applied out-of-plane directed in a ferro(i)magnet (FM), a spin voltage is generated via magnetization dynamics, pumping an out-of-plane spin current which flows into an attached normal (non-FM) metal (NM). As a next step, the spin current is converted into an electric field in the NM due to the inverse spin Hall effect (ISHE). The spin current generation parallel to the temperature gradient is the well-known longitudinal SSE (LSSE).

In 2010, Uchida et al. [6] exploiting the lack of free charge carriers in yttrium iron garnet (YIG) reported LSSE measurements on Pt/YIG. LSSE is then widely reproduced in different materials. Earlier publications have reported LSSE in FM insulators such as YIG [4,6–10], nickel ferrite [11–16], cobalt ferrite [17], magnetite [18–20] and FM semiconductor-like NiFe${\;}_{2}$O${\;}_{x}$ [21]. In addition, paramagnetic insulators [22], antiferromagnetics [23,24], noncollinear magnets [25] spin spirals [26] and topological insulators [27] have been investigated by LSSE. More recently, rather exotic materials such as one-dimensional spinon materials [28], graphene and other 2D layered materials [29,30] as well as altermagnets [31,32] have been in the focus of SSE studies.

The proximity of the NM to the FM can lead to an altering of their interfacial magnetic properties due to interfacial exchange coupling. Pt is usually employed for generating and detecting pure spin currents, however, due to its close vicinity to the Stoner criterion [33], the possibility of a magnetic proximity effect (MPE) should be carefully considered in transport measurements. MPE could significantly influence the spin transport properties and give rise to additional phenomena that do not exist in the constituent materials in isolation. Still, LSSE in magnetic insulators is free from parasitic contributions [34,35]. In accordance, most research groups have shown that the MPE in Pt on several insulators is not detectable [12,13,36–40]. For example for Pt/YIG, Kikkawa et al. provided an upper limit of 0.006 $\mu_{B}$ per Pt atom at 5.5 K [39] while Geprägs et al. demonstrated maximal 0.002 $\mu_{B}$ per Pt atom at room temperature [40]. However, FM metals (FMMs) or semiconducting-like FM [18,21,41] can exhibit an anomalous Nernst effect (ANE) [42,43] as well as a proximity-induced ANE [21,44] that prevent from the correct appraisal of the LSSE signal.

The exclusion of ANE contributions in Pt/FM bilayer systems is reported by Duan et al. [45] and Kannan et al. [46]. They varied Fe thickness and NiFe alloy composition, respectively, to achieve zero ANE contribution and thus studied the LSSE without parasitic effects. Any sign change observed with thickness variation is specific for exactly that composition and any sign change observed with composition variation is specific for exactly that thickness. Therefore, this approach cannot be used in general. Furthermore, Samransuksamer et al. analyzed the impact of Fe film thickness and Si(B) substrate on LSSE and ANE [47]. Yang et al. investigated the LSSE and ANE in Pt on the elementary materials Fe, Co, and Ni as well as permalloy [48].

Another technique to distinguish between LSSE and proximity-induced ANE was introduced by Kikkawa et al. [34,49]. In their analysis, the voltage measured transverse to the temperature gradient in in-plane magnetized (IPM) and out-of-plane magnetized (OPM) configurations results in the separation of the aforestated contributions. In our previous work [21,50], we extended this technique to identify all three contributions quantitatively: LSSE, ANE in the FM, and proximity-induced ANE in Pt on mainly insulating or badly conducting NiFe${\;}_{2}$O${\;}_{x}$ as well as on metallic Ni${\;}_{33}$Fe${\;}_{67}$. Now, we applied this procedure to tackle the issue of the full separation of the effects in two conducting NM/FMM bilayer series [50]. We investigated Pt/Co${\;}_{1-x}$Fe${\;}_{x}$ and Pt/Ni${\;}_{1-x}$Fe${\;}_{x}$ bilayers focusing on the correlation of the transport phenomena to the compositions and the magnetic moment of both the FMM and the spin-polarized Pt layer. This is interesting also because of the spin polarization at the Fermi level of these 3d metal alloys which is almost independent of the composition [51,52], an effect due to cancellation of density of states and Fermi velocity in the s and d bands.

The significant proximity-induced ANE contribution reported in this work and its dependence on the FMM and spin-polarized Pt layer magnetic moments unveils that MPE is a key element of spintronics and could modulate and emerge the functionality of future spintronic and spin caloritronic devices.

Up to this point, we generally described the SSE in terms of the generation of a spin voltage as a response to an applied temperature gradient. However, Bauer et al. [1] distinguished the spin current generation via spin accumulation of conduction electrons in FMM or FM semiconductors from magnon-based SSE. They termed the electron-based thermally induced spin transport spin-dependent Seebeck effect (SDSE) to separate it from the magnon-based SSE. In our work, we cannot distinguish between SSE and SDSE and, thus, we use the term SSE throughout the whole manuscript for both phenomena.

## 2. Experimental methods and theoretical background

We fabricated Pt/Co${\;}_{1-x}$Fe${\;}_{x}$ bilayers with $x_{\mathrm{Fe}}=0.00,0.15,0.30,0.50,0.67,1.00$ and Pt/Ni ${\;}_{1-x}$Fe${\;}_{x}$ films with $x_{\mathrm{Fe}}$ = 0.00,0.19,0.50,0.67,1.00, respectively, by dc magnetron sputter deposition on top of (001)-oriented MgO substrates at room temperature. The composition ratio between the individual 3d metals has been adjusted by the power of the individual sputter target which has been checked by x-ray fluorescence analysis. Twin FM layers were prepared without and with Pt in-situ deposited on top, by covering one FM layer with a mask to obtain the same deposition conditions for the FM in both samples. The Ar pressure during the deposition for all FM and Pt layers was equal to 2 $\times 10^{-3}\;\mathrm{mbar}$ and the base pressure was 3 $\times 10^{-9}\;\mathrm{mbar}$. The appropriate sputter parameters were adjusted after evaluating the x-ray fluorescence spectra to achieve the desired composition.

**Figure 1.**
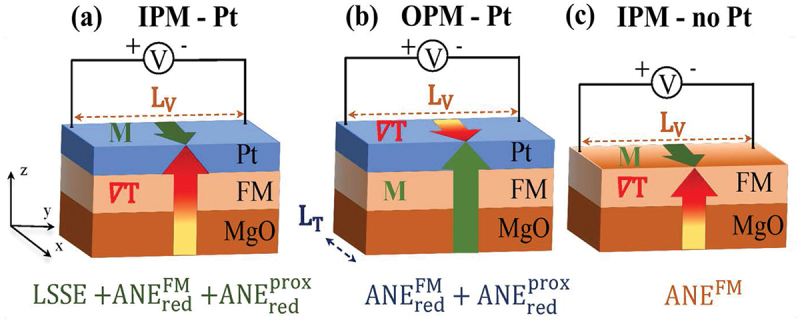
Schematic illustration of (a),(c) in-plane magnetized (IPM) and (b) out-of-plane magnetized (OPM) geometries, introducing the temperature gradient ∇T, the magnetization vector M, the distance between the contacts $L_{V}$, and the total length of the sample $L_{T}$, respectively. For each geometry the corresponding measured effect(s) is (are) denoted. Reproduced by permission from Ref. [21].

The measurement geometries employed for the quantitative separation of the effects are drawn in Figure 1. In the IPM geometries [Figure 1(a,c)] an out-of-plane temperature gradient ∇T is applied in the presence of an in-plane magnetic field along the x-axis and a transverse voltage is collected along the y-axis. Therefore, the sample is clamped between two copper blocks. One of them can be heated while the other acts as a heat sink. The temperature of each copper block is detected by a K-type thermocouple sensor. In addition, a Peltier element is placed between the substrate side of the sample and one copper block in order to detect the heat flux as described further below. Thermal grease has been used to ensure a good thermal contact between the copper blocks and the sample while a layer of aluminum oxide has been placed between one copper block and the Pt side of the sample to avoid electrical short circuits between the sample and the setup. The Pt layer is wire-bonded and the bonds are connected to copper wires in order to pick up the ISHE voltage. The measurements have been done under vacuum. Find more details and a picture of the sample holder in Refs. [50,53]. While measuring the samples with Pt on top in the IPM configuration [IPM – Pt, Figure 1(a)], we detect the LSSE voltage together with both ANE contributions, FM-induced and proximity-induced. In contrast, in the IPM geometry using samples without Pt on top [IPM – no Pt, Figure 1(c)], we are only sensitive to the ANE contribution from the FM.

The LSSE voltage is obtained by the ISHE, e.g.

(1) $$E_{\mathrm{ISHE}}\propto J_{s}\times s$$

for which $E_{\mathrm{ISHE}}$, $J_{s}$, and s indicate the electric field induced by ISHE, the spin current entering the spin detector material and the spin polarization vector, respectively. The spin current $J_{s}$ depends on the applied temperature gradient via the SSE coefficient $S_{\mathrm{SSE}}$. Furthermore, the ANE contribution is determined by the relation

(2) $$E_{\mathrm{ANE}}\propto \mathrm{\nabla T}\times M$$

for which $E_{\mathrm{ANE}}$ and M denote the electric field induced by ANE and the magnetization vector of the FM, respectively.

In the OPM geometry utilizing samples with Pt on top [OPM – Pt, Figure 1(b)], the transverse voltage is collected after the application of an in-plane temperature gradient ∇T in the presence of an out-of-plane magnetic field. This is attributed to the FM-induced and proximity-induced ANE. The LSSE signal is neglected in this configuration, since no out-of-plane spin current with the proper spin polarization direction is generated [34]. The in-plane temperature gradient is generated by placing the sample edges at the top of both copper blocks instead of clamping the top and bottom side of the sample between the blocks. Again, thermocouples are used to detect the temperatures at the copper blocks as well as a Peltier element is placed on the cold side between sample and copper block. As for the IPM configuration, thermal grease and wire bonding have been used. Further information is given in Ref. [50].

As explained in Ref. [21], it is crucial to consider the reduction of the ANE signal upon a placement of a Pt layer [18]. The generated transverse electric field that is built up by the ANE is shunted by the Pt layer. Therefore, the thickness of the Pt layer is an important parameter here and considered when calculating the non-reduced ANE electric field. For the nomenclature, all ANE signals measured with Pt on top will be denoted with the subscript ‘red’ in Figure 1 and throughout the whole manuscript, indicating their reduced contributions.

The analysis of the flow chart for the quantitative disentanglement of the three effects is included in our previous work and can be found in Ref. [21]. Summarizing the basic steps, the electric field is calculated from the measured voltage by normalizing it to the distance of the electric contacts $L_{V}$. Then, it is normalized to the heat flux $\Phi_{q}$ that runs through the sample. The advantageous employment of the heat flux, as suggested by Sola et al. [10,53,54], is focused on its independence from the interface thermal resistances and thermal contacts [55] allowing for an effective comparison between IPM and OPM configurations as well as for the comparability of our results.

The heat flux is estimated by using Peltier elements as heat flux sensors which have been calibrated using a 100 Ω resist as explained in Ref. [10]. Thus, the heat flux can be directly measured for the IPM configuration when the Peltier element is placed between the substrate and one copper block. Here, the cross section A between Peltier element and sample has to be taken into account when calculating the heat flux $\Phi_{q}=\frac{Q}{A}$ with the heat Q obtained by the calibrated Peltier element. For the OPM configuration, the Peltier element is placed between the cold sample side and the copper block. Here, the thermal conductivities of the individual layers have to be taken into account. Since the MgO substrate has the major contribution here compared to the thin films, its thermal conductance $K_{\mathrm{MgO}}=30\;Wm^{-1}K^{-1}$ determines the in-plane temperature gradient $\mathrm{\Delta T}=\frac{Q\;L_{T}}{K_{\mathrm{MgO}}\;S}$ with $L_{T}$ being the length of the sample (see Figure 1) and S the cross section of the substrate. Assuming a fixed in-plane ΔT for all layers, the heat flux of the FM layer in the OPM configuration can be calculated by $\Phi_{q}=\frac{K\;\Delta T}{L_{T}}$ with the thermal conductance K of the metallic layer estimated by the Wiedemann-Franz law.

To determine the reduction of the pure FM-induced ANE ($\mathrm{AN}E^{\mathrm{FM}}$) due to the additional Pt layer, the correction factor A should be introduced within the equation $\mathrm{AN}E^{\mathrm{FM}}=A\cdot \mathrm{AN}E_{red}^{FM}$, with $\mathrm{AN}E_{red}^{FM}$ being the reduced $\mathrm{AN}E^{\mathrm{FM}}$. In particular, A is given by the formula $A=\frac{r+1}{r}$, for which r is the ratio of conductances G of the FM and the Pt in a parallel arrangement extracted as follows [18,21]

(3) $$r=\frac{G_{\mathrm{FM}}}{G_{\mathrm{Pt}}}=\frac{\rho_{\mathrm{Pt}}}{\rho_{\mathrm{FM}}}\frac{t_{\mathrm{FM}}}{t_{\mathrm{Pt}}}$$

with ρ being the room temperature resistivity and t the thickness of the corresponding layer. Furthermore, in order to extract the pure proximity-induced ANE ($\mathrm{AN}E^{\mathrm{prox}}$) signal, an additional correction factor has to be applied to the reduced proximity-induced ANE signal because of $\mathrm{AN}E^{\mathrm{prox}}=B\cdot \mathrm{AN}E_{red}^{\mathrm{prox}}$, due to the additional non-magnetic Pt layer. However, during the data processing, the A has already been applied on $\mathrm{AN}E^{\mathrm{prox}}$ in order to subtract the ANE ${\;}^{\mathrm{FM}}$ contribution (see Figure 1) and, thus, it has to be taken back by the factor 1/A. The additional correction factor B is given by $B=\frac{t_{Pt}^{SP}+t_{Pt}^{NM}}{t_{Pt}^{SP}}$, for which $t_{Pt}^{SP}$ and $t_{Pt}^{NM}$ are the thicknesses of the spin-polarized Pt layer and the non-magnetic fraction, respectively. The effective spin-polarized Pt thickness can be determined by x-ray resonant magnetic reflectivity (XRMR) [12,56]. Finally, the corrected $\mathrm{AN}E^{\mathrm{prox}}$ is given by the formula $\mathrm{AN}E^{\mathrm{prox}}=(B/A)\cdot A\cdot \mathrm{AN}E_{red}^{\mathrm{prox}}$. Further details for the aforementioned analysis can be found in Refs. [21,50]. It should be noted that thermoelectric effects might have an anisotropy which could affect the presented separation procedure. However, this effect of anisotropy would be small due to the cubic crystal structure of most of the 3d metal alloys investigated here.

Table 1 summarizes the measured physical parameters of all samples. Here, $t_{\mathrm{FM}}$ is the thickness of the FM, $t_{\mathrm{Pt}}$ is the thickness of the total Pt layer, $t_{Pt}^{NM}$ is the thickness of the non-magnetic fraction of Pt, $t_{Pt}^{SP}$ is the thickness of the spin-polarized fraction of Pt, $\rho_{\mathrm{FM}}$ is the electrical resistivity of the FM (measured on the samples without Pt on top), and $\rho_{\mathrm{Pt}}$ is the electrical resistivity of Pt, for each film, respectively. The $\rho_{\mathrm{Pt}}$ values were calculated from the measured ρ values of the twin samples with and without the Pt layer on top. For the determination of the correction factor A, the same resistivity $\rho_{\mathrm{Pt}}$ was used for the polarized and unpolarized fraction of the Pt layers. For the Pt/Co${\;}_{1-x}$Fe${\;}_{x}$ series, the FM and Pt thicknesses are taken from XRMR data of Ref. [57] whereas for the Pt/Ni${\;}_{1-x}$Fe${\;}_{x}$, the thicknesses are obtained via x-ray reflectivity measurements.

**Table 1.** Thickness of the FM ($t_{\mathrm{FM}}$), total Pt ($t_{\mathrm{Pt}}$), non-magnetic Pt ($t_{Pt}^{NM}$), and spin-polarized Pt ($t_{Pt}^{SP}$) layers.

Film	$t_{\mathrm{FM}}$ [nm]	$t_{\mathrm{Pt}}$ [nm]	$t_{Pt}^{NM}$ [nm]	$t_{Pt}^{SP}$ [nm]	$\rho_{\mathrm{FM}}$ [Ω m]	$\rho_{\mathrm{Pt}}$ [Ω m]	Pt moment ($\mu_{B}$/Pt atom)
Pt/Fe	10.0	3.0	2.0	1.0	$3.30\cdot 10^{-7}$	$8.10\cdot 10^{-8}$	0.60 ± 0.10
Pt/Co${\;}_{33}$ Fe${\;}_{67}$	9.6	3.2	2.0	1.2	$6.11\cdot 10^{-7}$	$8.05\cdot 10^{-8}$	0.72 ± 0.03
Pt/Co${\;}_{50}$ Fe${\;}_{50}$	8.2	3.1	1.9	1.2	$4.31\cdot 10^{-7}$	$8.01\cdot 10^{-8}$	0.71 ± 0.03
Pt/Co${\;}_{70}$ Fe${\;}_{30}$	9.9	3.0	1.8	1.2	$4.50\cdot 10^{-7}$	$8.11\cdot 10^{-8}$	0.76 ± 0.03
Pt/Co${\;}_{85}$ Fe${\;}_{15}$	10.0	3.0	1.8	1.2	$4.73\cdot 10^{-7}$	$8.13\cdot 10^{-8}$	0.49 ± 0.03
Pt/Co	9.8	2.9	1.6	1.3	$4.93\cdot 10^{-7}$	$8.18\cdot 10^{-8}$	0.43 ± 0.03
Pt/Ni${\;}_{33}$ Fe${\;}_{67}$	9.8	3.0	2.0	1.0	$5.86\cdot 10^{-7}$	$8.08\cdot 10^{-8}$	0.44 ± 0.10
Pt/Ni${\;}_{50}$ Fe${\;}_{50}$	10.0	3.0	2.0	1.0	$6.53\cdot 10^{-7}$	$8.51\cdot 10^{-8}$	0.35 ± 0.10
Pt/Ni${\;}_{81}$ Fe${\;}_{19}$	9.9	3.0	2.0	1.0	$4.42\cdot 10^{-7}$	$8.38\cdot 10^{-8}$	0.22 ± 0.10
Pt/Ni	9.9	3.0	2.0	1.0	$4.08\cdot 10^{-7}$	$8.21\cdot 10^{-8}$	0.08 ± 0.08

Room-temperature resistivity of the FM ($\rho_{\mathrm{FM}}$), Pt ($\rho_{\mathrm{Pt}}$) layers, and the Pt magnetic moment extracted from the XRMR measurements [12,57,58], for all samples, respectively.

## 3. Results and discussion

**Figure 2.**
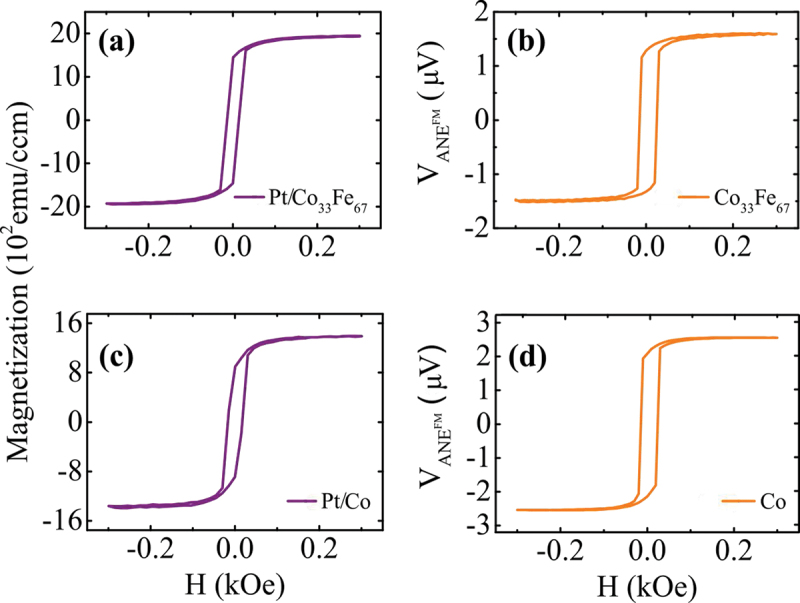
FM magnetization extracted from AGM measurements for (a) Pt/Co${\;}_{33}$Fe${\;}_{67}$ and (c) Pt/Co bilayers, respectively. $\mathrm{AN}E^{\mathrm{FM}}$ collected in the IPM – no Pt configuration for (b) Co${\;}_{33}$Fe${\;}_{67}$ and (d) Co single layers, respectively.

The magnetic properties of the Pt/Co${\;}_{1-x}$Fe${\;}_{x}$ were investigated by alternating gradient magnetometry (AGM) in a Princeton MicroMag using a magnetic field up to 1.3 T. The magnetization of the FM was extracted by hysteresis loops as indicatively presented in Figure 2(a,c) for the Pt/Co${\;}_{33}$Fe${\;}_{67}$ and Pt/Co bilayers, respectively. Figure 2(b,d) exhibit the corresponding voltage extracted from the measurements in the IPM – no Pt geometry, which is attributed to the $\mathrm{AN}E^{\mathrm{FM}}$ signal. In both cases, there is an obvious similarity in shape between the magnetic curve and the voltage curve, reflecting that the observation of the ANE signal is closely related to the magnetic stimuli.

**Figure 3.**
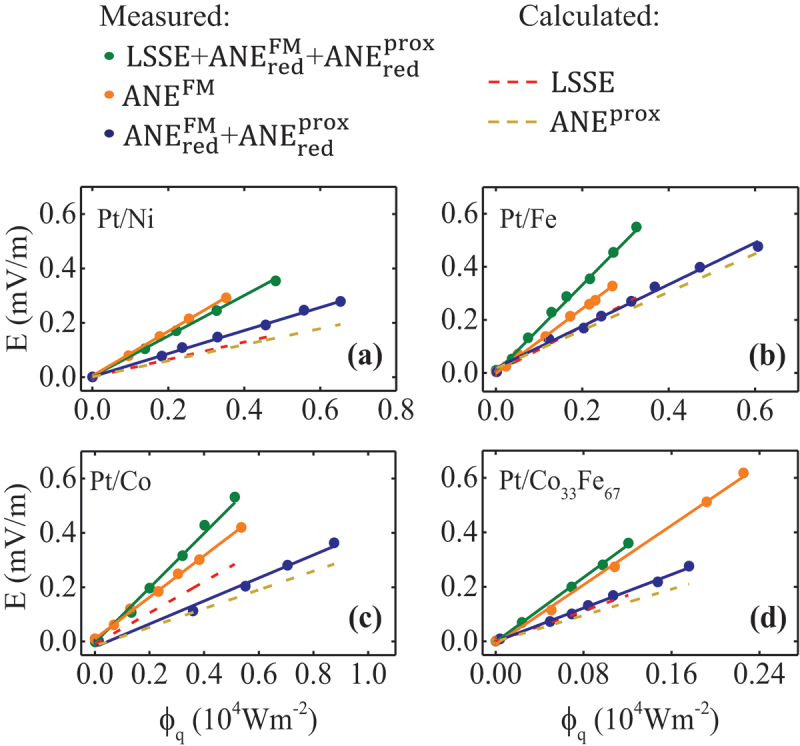
Electric field against the heat flux for (a) Pt/Ni, (b) Pt/Fe, (c) Pt/Co, and (d) Pt/Co${\;}_{33}$Fe${\;}_{67}$ samples with the corresponding separation of the ANE contribution (FM-induced and proximity-induced) from the LSSE signal.

Figure 3 illustrates the linear dependence of the electric field (voltage in saturation normalized to the electric contacts distance $L_{V}$) on the heat flux $\Phi_{q}$, for indicatively the Pt/Ni [Figure 3(a)], Pt/Fe [Figure 3(b)], Pt/Co [Figure 3(c)], and Pt/Co${\;}_{33}$Fe${\;}_{67}$ [Figure 3] bilayers. The linear dependencies have been extracted after evaluating the loop measurements (electric field vs. magnetic field for a series of thermal fluxes) collected in the IPM – Pt, IPM – no Pt, and OPM – Pt configurations, for all sample series. The green points depict the signal collected in the IPM – Pt configuration which includes the LSSE contribution as well as the reduced ANE signals (LSSE+ANE${\;}_{red}^{FM}$+ANE${\;}_{red}^{FM}$). The orange points exhibit the $\mathrm{AN}E^{\mathrm{FM}}$ contribution measured in the IPM – no Pt configuration. The blue points concern the measured signal from the OPM – Pt configuration, including both of the reduced ANE contributions (ANE ${\;}_{red}^{FM}$, ANE ${\;}_{\mathrm{prox}}^{FM}$). The dashed lines represent the calculated contributions of the pure LSSE (red) and $\mathrm{AN}E^{\mathrm{prox}}$ (yellow) signals, after considering the correction factors A and B for the reduced ANE signals, due to the spin-polarized and non-magnetic Pt layers, as introduced previously. The amplitudes of the error bars are too small to be resolved in this figure. The flow chart of the aforementioned analysis is described in Ref. [21]. The quantitative separation of the effects reveals that the $\mathrm{AN}E^{\mathrm{FM}}$ contribution dominates in all cases. In addition, the non-zero $\mathrm{AN}E^{\mathrm{prox}}$ signal indicates the existence of MPE for all samples confirmed by the Pt magnetic moments of Table 1 determined by XRMR [12,57,58].

**Figure 4.**
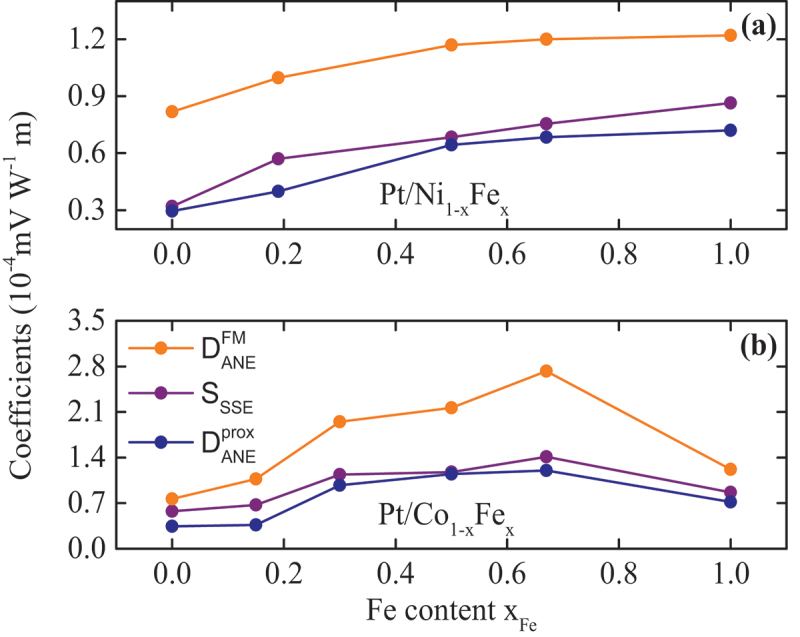
$\mathrm{AN}E^{\mathrm{FM}}$($D_{ANE}^{FM}$), sse ($S_{\mathrm{SSE}}$), and $\mathrm{AN}E^{\mathrm{prox}}$ ($D_{ANE}^{\mathrm{prox}}$) coefficients as a function of the Fe content $x_{\mathrm{Fe}}$ in the (a) Pt/Ni${\;}_{1-x}$ Fe ${\;}_{x}$ and (b) Pt/Co${\;}_{1-x}$Fe${\;}_{x}$ sample series. The error bars are smaller than the size of each dot.

Figure 4 depicts the dependence of the $\mathrm{AN}E^{\mathrm{FM}}$ ($D_{ANE}^{FM}=\frac{E_{ANE}^{FM}}{\Phi_{q}}$), SSE ($S_{\mathrm{SSE}}=\frac{E_{\mathrm{SSE}}}{\Phi_{q}}$), and $\mathrm{AN}E^{\mathrm{prox}}$ ($D_{ANE}^{\mathrm{prox}}=\frac{E_{ANE}^{p\mathrm{rox}}}{\Phi_{q}}$) coefficients on the Fe content $x_{\mathrm{Fe}}$ for both Pt/Co${\;}_{1-x}$Fe${\;}_{x}$ and Pt/Ni${\;}_{1-x}$Fe${\;}_{x}$ sample series, extracted from the corresponding slopes of the curves in Figure 3. Focussing on the Pt/Ni${\;}_{1-x}$Fe${\;}_{x}$ sample series in Figure 4(a), there is a pronounced increase of all coefficients with increasing Fe content. The $\mathrm{AN}E^{\mathrm{FM}}$ is the dominant contribution and the $\mathrm{AN}E^{\mathrm{prox}}$ possesses the lowest values for all compositions. The strength of the SSE contributions can be found in between. This behaviour is consistent with the trend depicted in Figure 3(a,b).

Moreover, by comparing the concluded coefficients for the Pt/Ni${\;}_{33}$Fe${\;}_{67}$ bilayer with the results reported for the metallic sample in our previous work [21], we extract that $S_{\mathrm{SSE}}$ is comparable within the same order of magnitude whereas $D_{ANE}^{FM}$ is quite higher in this work. This observation can be attributed to the higher $\rho_{\mathrm{FM}}$ of the Ni${\;}_{33}$Fe${\;}_{67}$ layer fabricated in this work and could be interpreted as follows. Both the ANE and the anomalous Hall effect (AHE) in an FM involve the spin-dependent separation of charge carriers and, thus, they share the same origin of spin-orbit coupling. In other words, the ANE can be considered as the thermal counterpart of the AHE. It is already established that there are two mechanisms contributing to the AHE, the intrinsic mechanism and the extrinsic one (skew scattering and side jump) [59]. Both the intrinsic mechanism and the side jump obey the square relationship $\rho_{\mathrm{AHE}}\propto \rho_{FM}^{2}$, with $\rho_{\mathrm{AHE}}$ and $\rho_{\mathrm{FM}}$ corresponding to the AHE resistivity and the longitudinal resistivity, respectively [60]. Whereas, considering the skew scattering mechanism there is a linear dependence of $\rho_{\mathrm{AHE}}$ on $\rho_{\mathrm{FM}}$.

The ANE depends on the derivative of the AHE resistivity. The coeffcient $S_{\mathrm{xy}}$ reflects the ANE contribution and depends on the thermo-electric coefficient $\alpha_{\mathrm{xy}}$, the Seebeck coefficient $S_{\mathrm{xx}}$ and the electric conductivity $\sigma_{\mathrm{xx}}$. As explained for example by Pu et al. [61], $S_{\mathrm{xy}}$ can thus be expressed as $S_{\mathrm{xy}}=\frac{1}{\sigma_{\mathrm{xx}}}\;(\alpha_{\mathrm{xy}}-\sigma_{\mathrm{xy}}\;S_{\mathrm{xx}})$. Therefore, we expect higher ANE signals from samples with higher $\rho_{\mathrm{FM}}=1/\sigma_{\mathrm{xx}}$ values. In addition, Chuang et al. [62] reported an enhancement of ANE in FMs (Fe, Co, Ni) which is dominated by spin-orbit coupling through the intrinsic and side-jump mechanisms. The ANE is very sensitive to the details of the electronic band structure and an enhancement of the intrinsic or side-jump contribution could increase the ANE signal. However, more systematic research should be conducted examining samples with the same stoichiometry and thickness, while tuning the intrinsic and extrinsic contributions, by varying the electronic band structure and/or the level of defects.

For the Pt/Co${\;}_{1-x}$Fe${\;}_{x}$ bilayers in Figure 4(b), all coefficients increase with increasing Fe content peaking at the FM bilayer Pt/Co${\;}_{33}$Fe${\;}_{67}$. For Pt on pure Fe, all coefficients decrease. Similarly to the Pt/Ni${\;}_{1-x}$Fe${\;}_{x}$ sample series, the $\mathrm{AN}E^{\mathrm{FM}}$ is the dominant effect whereas the $\mathrm{AN}E^{\mathrm{prox}}$ has the lowest contribution of the three. Again, the strength of the SSE contributions can be found in between. These observations also verify the tendency sketched in Figure 3(c,d). It is worth noting that the dependence of all coefficients on the Fe content for both sample series is qualitatively the same as the FM and Pt moment dependence on the Fe content for the Pt/Ni${\;}_{1-x}$Fe${\;}_{x}$ [58] and Pt/Co${\;}_{1-x}$Fe${\;}_{x}$ [57] sample series, respectively.

**Figure 5.**
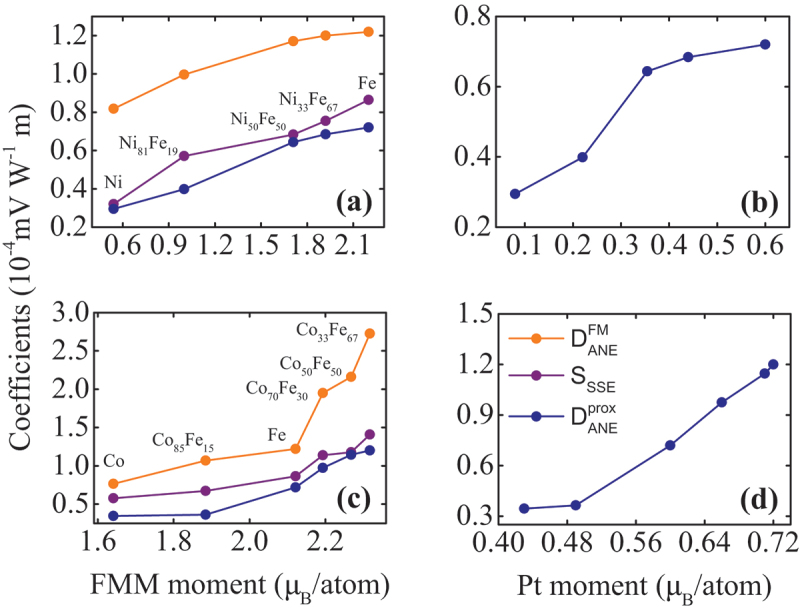
(a) Dependence of $D_{ANE}^{FM}$, $S_{\mathrm{SSE}}$, and $D_{ANE}^{\mathrm{prox}}$ on the FM moment and (b) $D_{ANE}^{\mathrm{prox}}$ dependence on the magnetic moment of the spin-polarized Pt layer for the Pt/Ni${\;}_{1-x}$Fe${\;}_{x}$ sample series. The FM and Pt moments are taken from Refs. [12,58] and the Pt moment for the Ni${\;}_{50}$Fe${\;}_{50}$ is interpolated data from Figure 8 of Ref. [58]. (c) Dependence of $D_{ANE}^{FM}$, $S_{\mathrm{SSE}}$, and $D_{ANE}^{\mathrm{prox}}$ on the FM moment and (d) $D_{ANE}^{\mathrm{prox}}$ dependence on the magnetic moment of the spin-polarized Pt layer for Pt/Co${\;}_{1-x}$Fe${\;}_{x}$ sample series. The FM moments are calculated data from the measured magnetization values on the same samples. The Pt magnetic moments are taken from Refs. [12,57].

Figure 5 exhibits the dependence of $D_{ANE}^{FM}$, $S_{\mathrm{SSE}}$, and $D_{ANE}^{\mathrm{prox}}$ on the corresponding FM and Pt magnetic moments. For the Pt/Ni${\;}_{1-x}$Fe${\;}_{x}$ series [cf. Figure 5(a)], all coefficients increase with increasing FM moment, similarly to the behaviour in Figure 4(a). Figure 5(b) presents the dependence of $D_{ANE}^{\mathrm{prox}}$ on the magnetic moment of the spin-polarized Pt layer extracted from XRMR measurements, for each alloy in the Pt/Ni${\;}_{1-x}$Fe${\;}_{x}$ series [58]. The error bars in the x-axis are included in Table 1 and left out from the graph for clarity reasons. The FM and Pt magnetic moments for the Pt/Ni${\;}_{1-x}$Fe${\;}_{x}$ series are taken from Refs. [12,58] and the Pt magnetic moment of the Pt/Ni${\;}_{50}$Fe${\;}_{50}$ bilayer is interpolated data from the data set in Figure 8 of Ref. [58]. The strong correlation between the magnetic moment of the Pt and the $\mathrm{AN}E^{\mathrm{prox}}$ is unveiled by the increase of $D_{ANE}^{\mathrm{prox}}$ with increasing Pt moment. For the Pt/Co${\;}_{1-x}$Fe${\;}_{x}$ bilayers [cf. Figure 5(c)], all coefficients increase with increasing FM magnetization, similarly to the Ni${\;}_{1-x}$Fe${\;}_{x}$ series. The FM moments are calculated data from the measured magnetization values on the same samples, assuming bcc structures for all compositions apart from the Co${\;}_{50}$Fe${\;}_{50}$ (fcc) and the Co (hcp) films. The Pt magnetic moments for the Pt/Co${\;}_{1-x}$Fe${\;}_{x}$ series are taken from Ref. [57] in which the XRMR data were collected on the same samples reported here, except from the Pt/Fe bilayer. For the Pt/Fe sample the magnetic moment is taken from Ref. [12], for which a similar Pt/Fe sample was examined. Additionally, the increase of the Pt moment in the alloy results in the enhancement of $\mathrm{AN}E^{\mathrm{prox}}$, as observed in Figure 5(d). Similarly to Figure 5(b), the error bars in the x-axis are left out from the graph, but can be looked up in Table 1.

According to Eq. (2), an increase in the magnetic moment in the FM (Pt) layer would imply an enhancement of the measured electric field of the $\mathrm{AN}E^{\mathrm{FM}}$ ($\mathrm{AN}E^{\mathrm{prox}}$). Thereby, the extracted trend of increasing the $\mathrm{AN}E^{\mathrm{FM}}$ ($\mathrm{AN}E^{\mathrm{prox}}$) coefficient with increasing the FM (Pt) magnetic moment is the expected behaviour for both sample series. In addition, the same dependence has been reported by Srichandan et al. [63] when examining the thermoelectric power of Co${\;}_{1-x}$Fe${\;}_{x}$ thin films, in order to mention another thermo-magneto-electric example of this dependence. They found an increase in the thermoelectric power in absolute values with increasing Fe content, in a range of $x_{\mathrm{Fe}}=(30\;-80)\%$. In addition, Ramos et al. [64] reported a sign change in the transverse thermoelectric voltage of a Co${\;}_{40}$Fe${\;}_{60}$/YIG bilayer, attributed to the presence of an interface-driven ANE due to sd-type exchange at the interface. According to their interpretations, at $x_{\mathrm{Fe}}=70\%$ as well as at other contents close to this value, we should be able to detect this sign change. However, in our ANE measurements, we do not see a sign change in the Co${\;}_{33}$Fe${\;}_{67}$ film.

Considering Eq. (1), a potential enhancement of the spin current owing from the FM to the Pt layer could induce a large electric field attributed. Under a thermal bias, there is a non-equilibrium accumulation at the NM/FM interface which pump the spin current into the NM. The pumped spin current is then converted into a charge current in the spin detecting NM. An explanation could be that the higher magnetic moment in the FM may indicate a higher magnetic density at the NM/FM interface which would imply a subsequent increase of the spin Seebeck coefficient due to a change in the spin mixing conductance [65]. Furthermore, the extracted $S_{\mathrm{SSE}}$ for the Pt/Ni${\;}_{33}$Fe${\;}_{67}$ bilayer is within the same order of magnitude compared to our previous work [21] and to the values reported by Rastogi et al. [16] on NFO/MgO samples and by Prakash et al. [55] on Pt/YIG films.

## 4. Conclusion

We investigated the thermal spin transport phenomena on sputter-deposited Pt/Co${\;}_{1-x}$Fe${\;}_{x}$ and Pt/Ni${\;}_{1-x}$Fe${\;}_{x}$ bilayers, reporting the quantitative separation of the LSSE signal from the ANE contributions which are induced by the FM and the spin-polarized Pt layer. For both sample series we extracted that the $\mathrm{AN}E^{\mathrm{FM}}$ is the dominant contribution and the $\mathrm{AN}E^{\mathrm{prox}}$ possesses the lowest values for all compositions. The strength of the SSE contributions could be found in between. In addition, we examined the SSE, $\mathrm{AN}E^{\mathrm{FM}}$, and $\mathrm{AN}E^{\mathrm{prox}}$ dependence on the composition as well as on the magnetic moment of both the FM and spin-polarized Pt layers. For the Pt/Ni${\;}_{1-x}$Fe${\;}_{x}$ series, all effect coefficients increase with increasing the Fe content in the films. For the Pt/Co${\;}_{1-x}$Fe${\;}_{x}$ series, the effect coefficients increase peaking at the composition Co${\;}_{33}$Fe${\;}_{67}$ whereas for the bilayer with the pure Fe, there is a pronounced drop. Similar observations have been reported in earlier investigations examining the evolution of the thermoelectric power with the composition in Co${\;}_{1-x}$Fe${\;}_{x}$ layers. We further extracted that all coefficients for both sample series present a qualitative analogy to the magnetic moment of both the FM and the spin-polarized Pt layers, pointing towards the direct connection between the magnetic moment of the films and the aforementioned effects. The increase of the SSE coefficient with increasing the FM moment in the alloy could be attributed to the higher magnetic moment density at the NM/FM interface, affecting the spin-mixing conductance and, thus, the SSE coefficient.
